# Identification of three novel *FGF16* mutations in X-linked recessive fusion of the fourth and fifth metacarpals and possible correlation with heart disease

**DOI:** 10.1002/mgg3.81

**Published:** 2014-05-14

**Authors:** Tobias Laurell, Daniel Nilsson, Wolfgang Hofmeister, Anna Lindstrand, Nadav Ahituv, Julia Vandermeer, Anders Amilon, Göran Annerén, Marianne Arner, Maria Pettersson, Nina Jäntti, Hans-Eric Rosberg, Peter A Cattini, Agneta Nordenskjöld, Outi Mäkitie, Giedre Grigelioniene, Ann Nordgren

**Affiliations:** 1 Department of Molecular Medicine and Surgery and Center of Molecular Medicine, Karolinska InstitutetStockholm, Sweden; 2 Department of Clinical Science and Education, Södersjukhuset, Karolinska InstitutetStockholm, Sweden; 3 Department of Hand Surgery, SödersjukhusetStockholm, Sweden; 4 Department of Clinical Genetics, Karolinska University HospitalStockholm, Sweden; 5 Science for Life Laboratory, Karolinska Institutet Science ParkStockholm, Sweden; 6 Department of Bioengineering and Therapeutic Sciences, University of California San FranciscoSan Francisco; 7 Institute for Human Genetics, University of California San FranciscoSan Francisco; 8 Department of Hand Surgery, Örebro University HospitalÖrebro, Sweden; 9 Department of Immunology Genetics and Pathology Science for Life Laboratory, Uppsala UniversityUppsala, Sweden; 10 Department of Clinical Sciences Malmö Section of Hand Surgery, Lund UniversityMalmö, Sweden; 11 Department of Hand Surgery, Skåne University HospitalMalmö, Sweden; 12 Department of Physiology, University of ManitobaCanada; 13 Department of Women's and Children's Health and Center of Molecular Medicine, Karolinska InstitutetStockholm, Sweden; 14 Unit of Paediatric Surgery Astrid Lindgren Children's Hospital, Karolinska University HospitalStockholm, Sweden; 15 Folkhälsan Institute of GeneticsHelsinki, Finland

**Keywords:** *FGF16*, heart, metacarpal fusion, MF4

## Abstract

Nonsense mutations in *FGF16* have recently been linked to X-linked recessive hand malformations with fusion between the fourth and the fifth metacarpals and hypoplasia of the fifth digit (MF4; MIM#309630). The purpose of this study was to perform careful clinical phenotyping and to define molecular mechanisms behind X-linked recessive MF4 in three unrelated families. We performed whole-exome sequencing, and identified three novel mutations in *FGF16*. The functional impact of *FGF16* loss was further studied using morpholino-based suppression of *fgf16* in zebrafish. In addition, clinical investigations revealed reduced penetrance and variable expressivity of the MF4 phenotype. Cardiac disorders, including myocardial infarction and atrial fibrillation followed the X-linked *FGF16* mutated trait in one large family. Our findings establish that a mutation in exon 1, 2 or 3 of *FGF16* results in X-linked recessive MF4 and expand the phenotypic spectrum of *FGF16* mutations to include a possible correlation with heart disease.

## Introduction

The fibroblast growth factor family (*FGF*) consists of 22 genes (Itoh and Ornitz 2004). FGFs are essential for diverse functions, mainly in development and metabolism, but also for limb bud formation and growth (Martin 1998; Mariani et al. 2008; Itoh and Ohta 2013). *FGF16* belongs to the paracrine *FGF-9* subfamily along with *FGF9* and *FGF20* and encodes a protein that is 207 amino acids long. FGF16 is a paracrine growth factor that until the recent report by Jamsheer et al. (2013) has not been associated with any human function or in mutated form, disease phenotype. *Fgf16* is mainly expressed in the heart, neural tube, and brown adipose tissue in rats and in the heart in mice (Konishi et al. 2000). Embryologic heart development in mice requires *Fgf16* and mice lacking *Fgf16* manifest myocardial hypoplasia, thinning of cardiac muscle, and dilatation of heart chambers (Lu et al. 2008a, 2010). Knockdown of *fgf16* in zebrafish with morpholinos (MOs) severely impairs outgrowth of fin buds, but has previously not been recorded with a cardiac phenotype (Nomura et al. 2006). Recently, Jamsheer et al. (2013) described for the first time two unrelated patients, one with inherited and one with a de novo nonsense mutation in *FGF16* on chromosome Xq21 as the causes of fusion between the fourth and the fifth metacarpals and hypoplasia of the fifth digit (MF4 MIM#309630). Jamsheer et al. (2013) also performed whole-mount in situ hybridization in mouse and observed a diffuse expression pattern of *Fgf16* in superficial mesenchymal cell layers in interdigital areas in the fore- and hindlimb.

MF4 (MIM#309630) has previously been described as isolated entities or as part of more complex hand malformations (Miura 1988; Ogino and Kato 1993). The hypoplastic small digit is often fixed in abduction. The inability to adduct the small digit can cause functional problems and motivates surgery (Ogino and Kato 1993; Ueba and Seto 1997). In 10 previously reported families isolated MF4 followed an X-linked recessive inheritance pattern (Orel 1928; Habighorst and Albers 1965; Holmes et al. 1972; Deliss 1977; Hooper and Lamb 1983; Anneren and Amilon 1994; Donovan et al. 1996; Lonardo et al. 2004; Kakish et al. 2011; Jamsheer et al. 2013) with the exception of one affected female in one family (Habighorst and Albers 1965). One family with autosomal dominant inheritance of MF4 (Lerch 1948) and 33 apparently de novo cases have been reported (Miura 1988; Kawabata et al. 1997; Foucher et al. 2001; Debeer et al. 2004; Havenhill et al. 2005; Yuan et al. 2010). Eleven of the sporadic cases were boys and in 22 cases gender was not reported. In 114 MF4 cases, family history has not been reported (Fm 1961; Buckwalter et al. 1981; Buck-Gramcko and Wood 1993; Ogino and Kato 1993; Ueba and Seto 1997; Gottschalk et al. 2012). Here we report three unrelated families with novel mutations in *FGF16*. We show that the severity of MF4 varies between family members and in one family that an early nonsense mutation in *FGF16* may have a correlation with human heart disease.

## Material and Methods

### Subjects

Three unrelated families with X-linked recessively inherited MF4 were included in the study. Families 1 and 3 originated from Sweden and family 2 from South America. We obtained a written informed consent from all participants or their legal guardians. The Local Ethics committee at Karolinska Institutet, Stockholm, Sweden, approved the study. All patients living in Sweden were clinically evaluated and their clinical data were reviewed.

### Whole-exome sequencing

Whole-exome sequencing (WES) was used for the proband and his cousin in family 1 and the proband and his brother in family 3. Libraries for sequencing on Illumina HiSeq2000 (Illumina, San Diego, CA) were prepared from DNA samples and exome sequences enriched with Agilent SureSelect Human All Exon 50 mol/L (Agilent, Santa Clara, CA), according to the manufacturer's instructions. Post capture libraries were sequenced as 2 × 100 base pairs (bp) paired end reads on the Illumina sequencer. Reads were base-called using offline CASAVA (v 1.7; Illumina). Sample library preparation, sequencing, and initial bioinformatics up to base-calling and demultiplexing were performed at the Science for Life Laboratory, Stockholm. An in-house pipeline, freely available under a GPL license (http://github.com/dnil/etiologica) was used to process reads and arrive at candidate genes. Briefly, reads were mapped to the human reference genome (hg19) using Mosaik (v1.0.1388) (Michael Strömberg, unpubl. data, http://code.google.com/p/mosaik-aligner/). Duplicate read pairs were removed using Mosaik DupSnoop. Variants were called using the SAMtools package (v.0.1.18). (Li et al. 2009) These were quality filtered (*Q* ≥ 20), and annotated using ANNOVAR (version 2011 October 05) (Wang et al. 2010). Variants were further filtered using ANNOVAR to remove those found at a 1000 genomes (Abecasis et al. 2010) minor allele frequency (MAF) of 2% and above, as well as variants found in dispensable genes, truncated at a MAF of more than 1% in any 1000 genomes subpopulation, and variants not predicted damaging by PolyPhen2 (Adzhubei et al. 2010) (*P*_threshold_ of 0.7). Nonsynonymous variants, indels, and putative splice site variants were retained. Remaining variants present in related affected individuals, but not found in a local cohort of 265 individuals with unconnected indication, were shortlisted.

### Sanger sequencing of *FGF16*

Sanger sequencing of *FGF16* was performed with seven primer pairs for the coding region of *FGF16* (exon 1, exon 2, exon 3) and the 1500 bp upstream region of exon 2 (intron 1) in order to confirm the nonsense mutation c.361G>T in *FGF16* detected by WES and to perform segregation analysis in family 1. Sanger sequencing was also used to screen for mutations and perform segregation analysis in families 2–3. Primer sequences are available on request. We used reference sequence for all *FGF16* exons and surrounding regions from NCBI RefSeq NW_003871101.3. Polymerase chain reaction (PCR) was performed with Taq Platinum polymerase and standardized conditions on an Applied Biosystems (Stockholm, Sweden) 2720 Thermal Cycler. The sequencing reaction was carried out with primers used for PCR and Big dye terminator v3.1 cycle sequencing kit. After purification, sequencing was done on an ABI-3730 DNA Analyzer, and the sequence was read in Seqscape v2.5.

### Maintenance of zebrafish

Zebrafish were maintained on a 14-h day 10-h night cycle at the Karolinska Institutets zebrafish core facility situated in the faculty of comparative medicine. Embryos were produced via light-induced spawning of Tupfel longfin (TL) *danio rerio* and raised at 28°C following microinjection.

### Morpholino and messenger mRNA injections

For all knockdown experiments Morpholino (MO) Genetools LLC (Philomath, OR) synthesized phosphodiestermer oligonucleotides. Two non overlapping MOs blocking either translation or splicing of *fgf*16 RNA were designed against the start AUG (fgf16tbMO: CGAGAAATCCAGCCACCTCTGCCAT) or the exon 2/intron 2 splice junction (fgf16sb: TGCAGGAGTTTGACTTACCGACCCA), respectively. To suppress any potential off-target effects induced by some MOs due to p53 activation, a previously published MO targeting p53 was used.(Robu et al. 2007) MOs were re-suspended in sterile water at a concentration of 25 mg/mL and stored at 4°C. In order to ascertain miss-splicing of the pre-mRNA, RNA was extracted from uninjected TL or *fgf*16 MO injected TL embryos and cDNA synthesized using Superscript III First strand synthesis kit (Invitrogen, Stockholm, Sweden). The resulting cDNA primers (Forward primer: GGATTTTGGCCACCTGAAAG and reverse primer: TTTTAGTCCTGCTGCCTTCC) spanning the splice junction were designed and used to amplify a 363 bp PCR product from 3dpf zebrafish embryos. The resulting product was (Sanger) sequenced and the introduction of a frameshift nonsense mutation was confirmed. For all knockdown experiments MOs were diluted to the desired concentrations in nuclease-free water and loaded into separate needles before being injected into the yolk of one to two cell embryos. Embryos were anesthetized in Tricaine (Sigma, Stockholm, Sweden) and screened for a reduction in pectoral fins and abnormal gross morphology at 3–4 dpf. For the acquisition of representative images, live embryos were placed in 3% methycellullose and images were taken on Leica DFC 230 microscope using Leica application Suite v4.1.0 (Leica Microsystems, Heerbrugg, Switzerland). All injections were repeated at least twice and similar results were pooled after testing for homogeneity of experimental groups using *χ*^2^ test of homogeneity. The significance in difference of phenotypic penetrance was analyzed using the *χ*^2^ test.

## Results

### Clinical features

**Figure 1:**
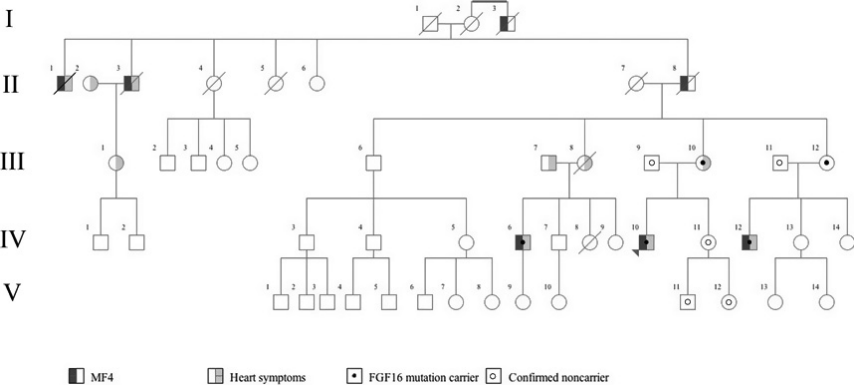
Pedigree of Family 1. The presence of MF4 is indicated by dark gray shading of the symbol's left side. Light gray shading of the symbol's right side indicates heart symptoms. Filled centered dot indicates verified *FGF16* mutation carrier and centered ring indicates verified nonmutation carrier.

**Figure 2:**
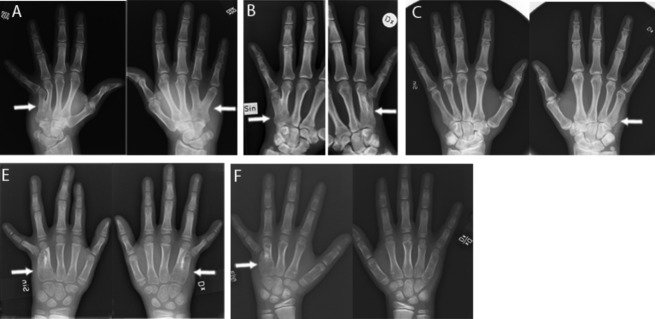
MF4 in families 1-3. Phenotype variability in MF4 in three cousins in family 1 with the c.361G>T (p.G121*) mutation (A–C). White arrows indicate fusion of the fourth and the fifth metacarpal in IV-10 (A), IV-12 (B) and a broader proximal part of the fifth metacarpal in IV-6 (C). White arrows also indicate fusion of the proximal part of the fourth and the fifth metacarpals in the probands of family 2 (E) and family 3 (F).

In the first family, previously described by Anneren and Amilon (1994) nine family members were available for testing. The pedigree showed an X-linked recessive inheritance pattern (Fig. 1). Clinical examination of the proband (IV-10) and his male cousin (IV-12) at the ages of 4 and 7 years showed bilateral ulnar deviation and hypoplasia of the fifth digits. Radiographs showed fusion between the fourth and fifth metacarpal (Fig. 2A). The fifth digits were fixed in 45° of ulnar deviation at the level of the metacarpophalangeal joint without active or passive adduction. Flexion and extension in the digits was normal. The maternal grandfather (II-8), his two brothers (II-1 and II-3), and their maternal uncle (I-3) had the same phenotype. Due to the dysfunctional position of the fifth digits individuals II-8, II-1, and I-3 had the fifth digits removed surgically. The proband's malformation in the left hand was surgically corrected at the age of 7. None of the family members reported any other malformations. The three male cousins (IV-6, IV-10, IV-12) who were later found to have a nonsense mutation in *FGF16* showed variable severity of MF4 (Fig. 2A–C). The least severely affected, IV-6, had a broad proximal fifth metacarpal bone and a minor overall shortening of the fifth digit.

Importantly, eight *FGF16* mutation-carrying members of family 1 reported cardiac symptoms. Five of these were proven heart disease (individual II-1, II-3, III-8, III-10, and IV-12) with myocardial infarction in four individuals and atrial fibrillation in one. The proband (IV-10) fainted at the age of 20 during an episode of severe chest pain. He was admitted to hospital but clinical examination was normal. Electrocardiogram (ECG), and echocardiography performed at the age of 38 years were normal. The smoking mother of the proband (III-10), who was heterozygous for the *FGF16* mutation, had a myocardial infarction at the age of 46 years. She underwent percutaneous coronary intervention twice but continues to have intermittent angina pectoris. Echocardiography at the age of 57 years and chest radiography at the age of 60 years revealed a mild mitral valve insufficiency and mild hypertrophy of the heart, respectively. One male cousin (IV-12) found later to have the *FGF16* mutation had a manifest atrial fibrillation requiring defibrillation at the age of 25 years. Echocardiography and 24-h ECG performed at the age of 36 years revealed a mild mitral valve insufficiency and short periods of sinus-tachycardia. A third male cousin (IV-6) who was also found to have the *FGF16* mutation sought health care due to recurrent episodes of heart palpitations at the age of 28 years, but ECG was normal. At the age of 35 years, ECG and echocardiography was normal. His mother (III-8), an obligate mutation carrier suffered from hypertension and died suddenly during sleep at the age of 55 years. The autopsy showed mild coronary arteriosclerosis, mild left-sided hypertrophy of the heart and a small healed myocardial infarction in the left inferior wall and concluded that cardiac arrhythmia could have been the cause of death. Two brothers of the proband's maternal grandfather (II-1 and II-3), reported to have the MF4 phenotype, died of myocardial infarction at the age of 73 and 51, respectively. His daughter (III-1) suffers from episodes of stress -related chest pains. At the age of 34, she was on hospital ward for tachycardia but recovered spontaneously. Unfortunately, she refused further genetic testing. None of the mutation negative family members had similar cardiac manifestations.

In family 2, only one affected 10-year-old boy and his parents were available for testing. The rest of the family lives in South America and could not be reached for this study. The pedigree shows an X-linked recessive inheritance pattern except for a female maternal cousin of the proband's mother that was reported to have the MF4 phenotype (Fig. S1). Clinical examination and radiographs of the proband at the age of 8 years showed bilateral MF4 with ulnar deviation and hypoplasia of the fifth digits (Fig. 2E). Two maternal uncles, one maternal uncle of the mother, two maternal cousins of the mother (one male and one female), and one male second cousin of the proband were reported to have the same phenotype. The proband was the only family member in whom corrective surgery had been performed. According to the patient's mother, none of the family members had any other malformation or cardiac symptoms. However, we have not been able to contact the family members in South America and can, therefore, not rule out the possibility of heart disease in these individuals.

In family 3, four family members were available for testing. The pedigree showed an X-linked recessive inheritance pattern (Fig. S2). Clinical examination of the proband and his brother at the age of 14 and 17 showed ulnar deviation and hypoplasia of the fifth digits. Radiographs showed a fusion of the proximal part of the fourth and the fifth metacarpals on the left side in the younger brother and right side in the older brother (Fig. 2F). No corrective surgery was done for the brothers or the maternal grandfather. None of the family members reported any other malformation or any cardiac symptoms.

### Mutation analyses using whole-exome and Sanger sequencing

To search for a disease-causing mutation in MF4, WES was performed on DNA samples from four affected individuals from family 1 and 3. In family 1, WES showed a c.361G>T (p.G121*) nonsense mutation at position chrX:76709734 (hg19) in exon 2 of *FGF16* in the proband and his male cousin. The c.361G>T (p.G121*) mutation results in a premature stop codon. The 42% of the FGF16 protein that does not get made contains the heparine-binding site of FGF16. Sanger sequencing confirmed the mutation in the proband and in two of his affected male cousins (Fig. S3). The proband's mother and one of his aunts were heterozygous for the mutation. The unaffected sister of the proband did not carry the mutation. The mutation was absent in WES of a local cohort of 265 individuals. In family 2, Sanger sequencing of *FGF16* in the proband showed a c.378G>C (p.S126S) transversion at position chrX:76709751(hg19) in the last nucleotide of exon 2 of *FGF16* (Fig. S3). The transversion was detected in a heterozygous state in the mother with Sanger sequencing. Guanine at position chrX:76709751(hg19) is highly conserved among different species (Fig. S4). The high degree of conservation and the position in the last nucleotide of exon 2 makes c.378G>C (p.S126S) likely to be pathogenic even though it was not predicted to significantly alter splicing according to Human splicing finder(Desmet et al. 2009) and NNSPLICE 0.9 version (January 1997). Unfortunately, no material was available for cDNA sequencing. The transversion was absent in WES of a local cohort of 265 individuals. In family 3 Sanger sequencing showed a c.203G>T transversion, leading to a change in positively charged argine to hydrophobic leucine at position 68 of the predicted amino acid sequence (p.R68L). In silico prediction with Panther Classification System, (http://www.pantherdb.org/tools/csnpScoreForm.jsp) (Brunham et al. 2005) indicated that the variant is possibly damaging with subPSEC at −3.57 and p deleterious 0.64. This Arginine at position 68 in FGF16 is highly conserved evolutionary (Fig. S5). Sanger sequencing did not detect the mutation in 88 unaffected control individuals. We were not able to check c.203G>T in the WES cohort since exon 1 of FGF16 is not annotated in hg19 and covered by the used capture kit (Human All Exon 50 mol/L from Agilent).

### Zebrafish MO experiments

**Figure 3:**
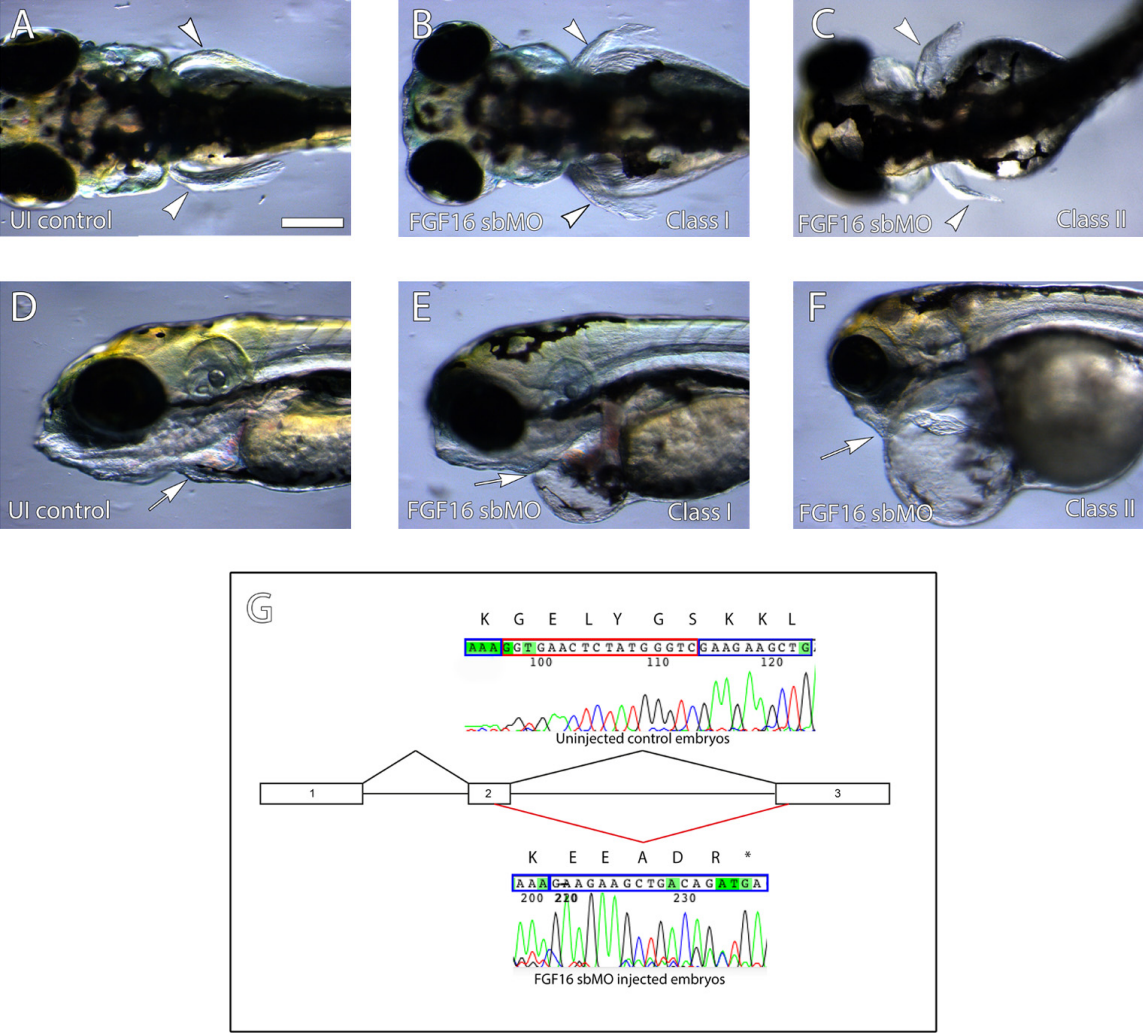
Knockdown of FGF16 results in reduced fins and heart malformations. Zebrafish embryos at 4 days post fertilization (dpf). Dorsal views of the head are shown in A–C and lateral views in E–F. Rostral is to the left in A–G. Injection of *fgf16* splice blocking morpholino (sbMO) results in a severe reduction in fins (filled arrowheads in B, C) compared to uninjected control embryos (A) and in addition, embryos injected with *fgf16* MO also show edema of the heart (filled arrow; E and F; sbMO shown) compared to uninjected embryos (E). Knockdown of the transcript was confirmed via sequencing of RT-PCR products (G). sbMO binding results in cryptic splice donor site leading to the deletion of 16 bp at the end of exon 2.

In order to mimic abnormal splicing of *fgf16* a splice blocking morpholino (sbMO) was designed to bind across the splice junction for exon2/intron3 of Zebrafish *fgf16*. Injection of 5 ng of *fgf16* sbMO resulted in a severe reduction in the size of the pectoral fins, designated here as a class I phenotype, in 46% of 101 embryos injected (Fig. 3). Our findings were similar to a previous report (Nomura et al. 2006) but we did not observe a complete absence of the fin buds as previously reported (Nomura et al. 2006). Thirty percent of the 101 embryos injected also showed a more severe gross malformation of the body in addition to the fin phenotype designated as class II phenotype (Fig. 3). Affected embryos also developed heart edema with 76% of the 101 injected embryos showing this phenotype. It is worth noting that this phenotype has been shown to be a potential off-target effect of MOs (Robu et al. 2007). Aberrant splicing of *fgf16* was confirmed by RT-PCR (Fig. 3 G). MO binding results in cryptic splice donor site leading to the deletion of 16 bp at the end of exon 2 (Fig. 3 G). This deletion results in a frameshift mutation leading to a premature truncation of the transcript and most likely nonsense-mediated decay. To ensure the observed phenotypes were due to the specific knockdown of *fgf16*, we designed a second MO against the translational start site to block ribosome binding. Injection of this translational blocking MO gave similar results (data not shown) including the presence of heart edema. As this is possibly an off-target effect due to p53 activation, we co-injected 5 ng *fgf16* MO with 7.5 ng of p53 MO, but no significant change in phenotype of the embryos was seen following co-injection with p53. Injection of wild-type (WT) *FGF16* mRNA alone, even at low concentrations, resulted in early gastrulation defects, most likely due to ubiquitous expression of FGF16 perturbing the endogenous expression gradient. Since the embryos did not survive to 24 hpf (data not shown) the WT rescue assay for fin or heart defects was not convincing and no mutant rescue was performed. Taken together these results confirm the role of *fgf16* in fin development of the zebrafish and suggest a potential link to heart development.

## Discussion

We have studied three unrelated families with X-linked recessive MF4 and as a result have: (1) identified three novel *FGF16* mutations that help establish this as the cause for X-linked recessive MF4; (2) shown the capacity for variable phenotype of MF4 among family members with mutations in *FGF16*; and (3) found possible correlation with heart disease.

In this study, we have identified three previously non-described variants in the *FGF16* gene in three unrelated cases with MF4: one nonsense mutation c.361G>T (p.G121*) in exon 2 and two likely pathogenic variants; c.203G>T (p.R68L) in exon 1 and c.378G>C in exon 2. The two previously reported nonsense mutations in the MF4-study of Jamsheer et al. (2013) (p.R179* and p.S157*) are located in exon 3. Jamsheer et al. (2013) suggest that the position of their nonsense mutations make the transcripts likely to be stable and escape nonsense-mediated decay. Therefore, the truncated proteins could still exert some residual activity during embryogenesis, which might prevent severe heart developmental defects as reported in *Fgf16* deficient mice (Lu et al. 2008a, 2010). As the p.G121X nonsense mutation in this study is positioned upstream to those mutations, in exon 2, the transcript is more likely to be a subject to nonsense-mediated RNA decay. If the transcript escapes nonsense-mediated decay it will be truncated before the heparin-binding site of FGF16, which may contribute to the pathogenicity of the mutation. The previously undescribed variant c.378G>C (p.S126S) in family 2 is located in a highly conserved DNA sequence in the last nucleotide of exon 2, one position upstream of the intron 2 splice donor site, which supports the possibility that this change might be pathogenic. The c.203G>T transversion in family 3 was predicted with Panther Classification System, (http://www.pantherdb.org/tools/csnpScoreForm.jsp) to be possibly damaging with subPSEC at −3.57 and p deleterious 0.64. Also c.203G>T might change the function or structure of FGF16 since it codes for hydrophobic leucine instead of positively charged arginine at the highly conserved position 68 of the FGF16 protein; this needs to be further investigated with functional studies.

The functional impact of the identified *FGF16* changes was confirmed using MO-based suppression of *fgf16* in zebrafish. In a previous *fgf16* knockdown study by Nomura et al. (2006) the fins of zebrafish are only visible as shallow domes at 72 h post fertilization (Nomura et al. 2006). In this study, the size of the fins were larger, approximately half the size of those of the controls, and more developed at 72–96 h post fertilization. Nomura et al. (2006) has further shown that *fgf16* knockdown inhibits *fgf4* and *fgf8* expression in the apical ectodermal ridge and Sonic Hedgehog (SHH) expression in the zone of polarizing activity on the postaxial side of the fin bud. Normally *fgf4* and *fgf8* are both expressed in the pectoral fin bud (Grandel et al. 2000) and double knockout of *Fgf4* and *Fgf8* in mice severely impairs limb development (Boulet et al. 2004).

Phenotype variability was observed among affected individuals in family 1 (Fig. 2A–C). For example, the least severely affected individual only has a broad proximal fifth metacarpal bone and an overall shorter fifth digit. The more severely affected individuals have undergone surgery due to dysfunctional positions with ulnarly deviated and hypoplastic fifth digits with fusion between the fourth and fifth metacarpal. In family 2 and in the report by Habighorst and Albers (1965), one female in each family had the MF4 phenotype. A probable explanation for the presence of symptoms in heterozygous female mutation carriers may be skewed X-chromosome inactivation.

*FGF16* has not been previously implicated in human heart disease, but it is known that embryonic heart development in mice requires *Fgf16* (Lu et al. 2008a). Interestingly, three individuals in family 1, carrying the nonsense mutation in *FGF16,* had myocardial infarction at an early age. (51, 46, 55 years) compared to the median age for 2012 in Sweden, 70–74 years in male and 80–84 years in female (National board of health and welfare: http://www.socialstyrelsen.se/statistics). One brother of the proband's maternal grandfather (II-1) died of myocardial infarction at the age of 51 and the mother of the proband (III-10) had a myocardial infarction at the age of 46. The maternal aunt of the proband (III-8) died at the age of 55 and autopsy showed signs of a previous myocardial infarction and the cause of death was cardiac failure. One of the cousins of the proband (IV-12) had a manifest atrial fibrillation requiring defibrillation at the age of 25. It is noteworthy that two family members showed mild hypertrophy of the heart (III-8 and III-10) even if it could be secondary to hypertension in case III-8.

In accordance with the cardiac manifestations in the *FGF16* nonsense mutation carrying family members, 76% of 101 zebrafish embryos injected in this study had a heart edema (Fig. 3). This was observed for both *fgf16* MOs used and was not rescued by co-injection with p53-MO. Further studies in a stable knockdown fish are required to clarify the role of fgf16 in zebrafish heart development.

Fgf16 might exert a function in adult mice since expression levels in the heart are more abundant at adult stages than at embryonic stages (Hotta et al. 2008; Lu et al. 2008b). To investigate the potential role of Fgf16 in the heart at adult stages Matsumoto et al. (2013) induced cardiac hypertrophy by injecting angiotensin II to a breed of *Fgf16*^−/−^ mice. The hearts of the *Fgf16*^−/−^ mice were more hypertrophic and fibrotic than in wild-type mice suggesting that Fgf16 prevents angiotensin II-induced cardiac hypertrophy and fibrosis (Matsumoto et al. 2013). Fgf16 has been described to prevent cardiac hypertrophy and fibrosis by competing with Fgf2 for the binding site of the FGF receptor 1c, Fgfr1c, in a paracrine manner (Itoh and Ohta 2013). Fgf2 promotes cardiac remodeling by activating mitogen-activated protein kinases signaling through the activation of Fgfr1c (Itoh and Ohta 2013; Sontag et al. 2013). Family 1 in our report is the only known family where frequent cardiac manifestations have occurred among mutation carrying family members. The two cases described by Jamsheer et al. (2013) both with a nonsense mutation, were not reported to have any heart-related symptoms. One may speculate that differences in location of the truncating mutation in exon 2 of our patients compared to those in exon 3 reported by Jamsheer et al. (2013) may explain the early-onset cardiac symptoms in family 1 and the absence of cardiac symptoms in the other cases. Alternatively, different mutations may affect the level of susceptibility of an individual to develop heart disease in response to lifestyle choices or another condition, like diabetes. There is also evidence in mice that the genetic background can modify the severity of the phenotype detected with Fgf16 knockout (Lu et al. 2008a, 2010). Another possibility is that cardiac symptoms could have escaped detection in previous reports that were focused on MF4.

In conclusion, this study not only establishes mutations in exons 1, 2 or 3 of *FGF16* as the cause of X-linked recessive MF4 but also suggests an association of *FGF16* nonsense mutation with cardiac disease. Further studies are needed to highlight pathogenic mechanisms of the *FGF16* mutations, and the possible correlation between *FGF16* mutations and cardiac disease needs to be explored in a larger number of patients.

## Supporting Information

**Figure S1.** Pedigree of family 2. Filled symbols indicate MF4. Red dots indicate verified mutation carriers.

**Figure S2.** Pedigree of family 3. Filled symbols indicate MF4. Red dots indicate verified mutation carriers.

**Figure S3.** Electropherogram of the mutations in families 1–3. Electropherogram showing the c.361G>T (p.G121*) nonsense mutation at position chrX:76709734 (hg19) in the proband (A), the heterozygous mother (B) and the healthy father (C) of family 1. In family 2 the c.378G>C synonymous mutation at position chrX:76709751(hg19) in the last nucleotide of exon 2 of *FGF16* is shown for the proband (D), the heterozygous mother (E) and the healthy father (F). The c.203G>T (p.R68L) missense mutation in exon 1 of FGF16, AC243316.3 of the proband and his brother in family 3 is shown in G.

**Figure S4**. Conservation for the position of c.378G>C in family 2. Guanine at position 378 in *FGF16*, chrX:76709751(hg19), is conserved among human, rhesus monkey, cow, mouse, rat, and hen, showing a high degree of conservation.

**Figure S5.** Conservation for the position of c.203G>T (p.R68L) in family 3. Arginine at position 68 in the FGF16 protein is conserved in human, megabat, cow, mouse, hen, and zebrafish. The c.203G>T (p.R68L) missense mutation of family 3 is, therefore, likely to alter function of FGF16.
