# Supplementary figures and images for: Identification of three novel FGF16 mutations in X-linked recessive fusion of the fourth and fifth metacarpals and possible correlation with heart disease
Source: Mol Genet Genomic Med. 2014 May 14;2(5):402–11. doi: 10.1002/mgg3.81 (PMC4190875; doi:10.1002/mgg3.81)

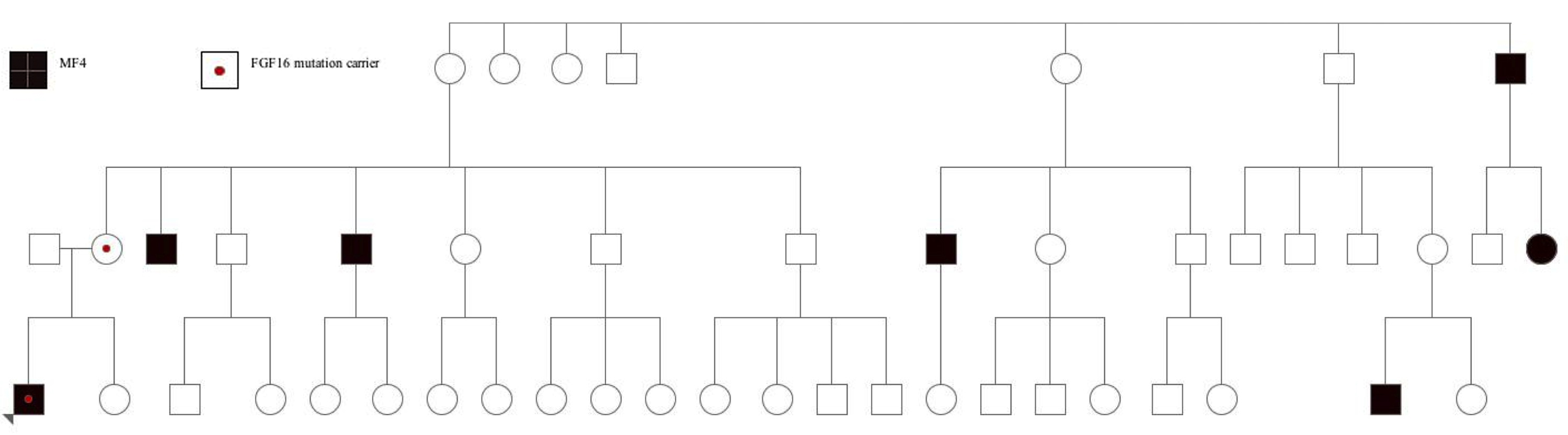

Supplement: Supplementary file 1 — Figure S1. Pedigree of family 2. Filled symbols indicate MF4. Red dots indicate verified mutation carriers. [file mgg30002-0402-SD1.tiff]

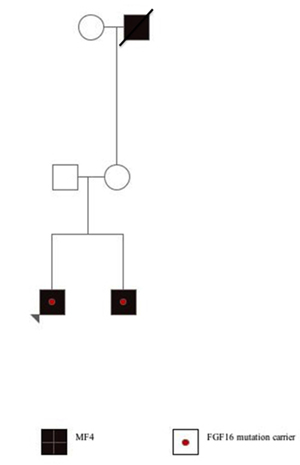

Supplement: Supplementary file 2 — Figure S2. Pedigree of family 3. Filled symbols indicate MF4. Red dots indicate verified mutation carriers. [file mgg30002-0402-SD2.tiff]

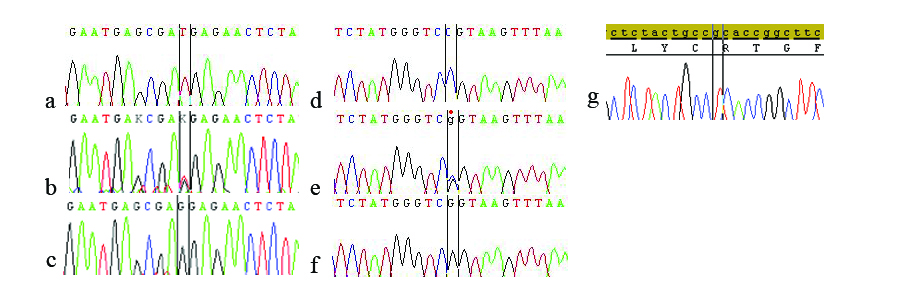

Supplement: Supplementary file 3 — Figure S3. Electropherogram of the mutations in families 1–3. Electropherogram showing the c.361G>T (p.G121*) nonsense mutation at position chrX:76709734 (hg19) in the proband (A), the heterozygous mother (B) and the healthy father (C) of family 1. In family 2 the c.378G>C synonymous mutation at position chrX:76709751(hg19) in the last nucleotide of exon 2 of FGF16 is shown for the proband (D), the heterozygous mother (E) and the healthy father (F). The c.203G>T (p.R68L) missense mutation in exon 1 of FGF16, AC243316.3 of the proband and his brother in family 3 is shown in G. [file mgg30002-0402-SD3.tiff]

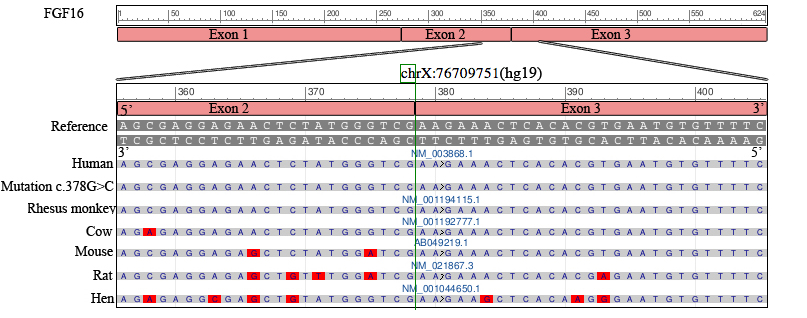

Supplement: Supplementary file 4 — Figure S4. Conservation for the position of c.378G>C in family 2. Guanine at position 378 in FGF16, chrX:76709751(hg19), is conserved among human, rhesus monkey, cow, mouse, rat, and hen, showing a high degree of conservation. [file mgg30002-0402-SD4.tiff]

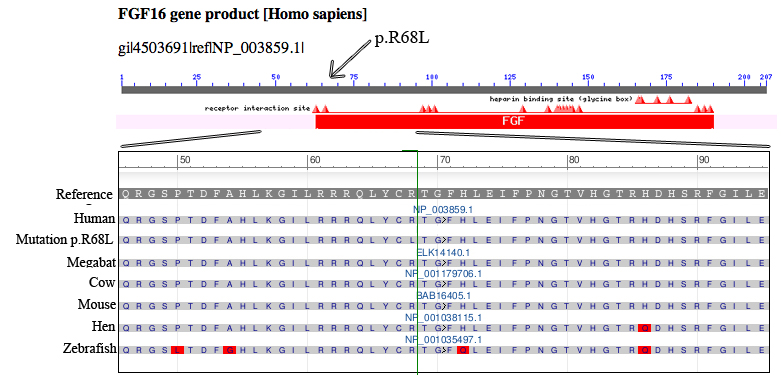

Supplement: Supplementary file 5 — Figure S5. Conservation for the position of c.203G>T (p.R68L) in family 3. Arginine at position 68 in the FGF16 protein is conserved in human, megabat, cow, mouse, hen, and zebrafish. The c.203G>T (p.R68L) missense mutation of family 3 is, therefore, likely to alter function of FGF16. [file mgg30002-0402-SD5.tiff]
